# A Review of the Epidemiological Evidence for Adducin Family Gene Polymorphisms and Hypertension

**DOI:** 10.1155/2019/7135604

**Published:** 2019-06-02

**Authors:** Jing rui Zhang, Wan ning Hu, Chang yi Li

**Affiliations:** ^1^Department of Cardiology, Beijing Anzhen Hospital, Capital Medical University, Beijing 100020, China; ^2^Laboratory of Molecular Biology, Tangshan People's Hospital, Tangshan 063000, China

## Abstract

Hypertension is one of the most common cardiovascular diseases that seriously endangers human health and has become a significant public health problem worldwide. In the vast majority of patients, the cause of hypertension is unknown, called essential hypertension (EH), accounting for more than 95% of total hypertension. Epidemiological and genetic studies of humans and animals provide strong evidence of a causal relationship between high salt intake and hypertension. Adducin is one of the important candidate genes for essential hypertension. Adducin is a heterodimeric or heterotetrameric protein that consists of *α*, *β*, and *γ* subunits; the three subunits are encoded by genes (*ADD1*, *ADD2*, and *ADD3*) that map to three different chromosomes. Animal model experiments and clinical studies suggest that changes in single-nucleotide polymorphisms (SNPs) at part of the adducin family gene increase the Na^+^-K^+^-ATPase activity of the renal tubular basement membrane and increase the reabsorption of Na^+^ by renal tubular epithelial cells, which may cause hypertension. This review makes a summary on the structure, function, and mechanism of adducin and the role of adducin on the onset of EH, providing a basis for the early screening, prevention, and treatment of EH.

## 1. Introduction

Hypertension, typically defined as a resting systolic BP (SBP) 140 mm·Hg or higher, or diastolic BP (DBP), 90 mm·Hg or higher, or receiving therapy for the indication of BP-lowering, afflicts a substantial proportion of the adult population worldwide [1, 2] and leads to cardiovascular events (some of which, such as stroke, myocardial infarction, sudden death, heart failure, and peripheral artery disease), as well as end-stage renal disease [3]. Numerous studies have examined potential genetic susceptibilities for hypertension [4, 5]. Current genetic epidemiological studies suggest that adducin may be the susceptible gene of essential hypertension (especially salt-sensitive EH) [6–11]. Hence, the association between adducin gene polymorphisms and EH has been widely concerned. Herein, we describe the structure and function of adducin, summarize the current knowledge on the relationship between adducin family gene polymorphisms and EH, and propose a more comprehensive approach to the prevention and treatment of EH.

## 2. Structure of Adducin

Adducin is a ubiquitously expressed cytoskeleton protein, which was initially been purified from human erythrocytes by Gardner and Bennett in 1986 [12] and was soon separated from bovine brain cells. In 1991, Joshi et al. found two subunits of adducin termed *α*-adducin (*ADD1*) and *β*-adducin (*ADD2*), respectively [13]. Dong et al. in 1995 reported that adducin still had *γ* subunits (*ADD3*) [14]. The three subunits are produced from distinct genes but share a similar structure [14–17]. All the three adducin proteins contain an N-terminal globular head domain, a neck domain and a C-terminal protease-sensitive hydrophilic C-tail domain [18]. At the end of the tail domain, there is a 22-residue MARCKS-related domain that has high homology to myristoylated alanine-rich C kinase substrate (MARCKS) protein. The MARCKS-related domain has clusters of lysine residues and is highly conserved among the three adducin subunits. *α*- and *γ*-adducins are ubiquitously expressed. In contrast, *β*-adducin is expressed at high levels in the brain and hematopoietic tissues.

Human *ADD1* is localized at chromosome 4p16.3 [19], the spans of which was about 85 kb and contained 16 exons ranging in size from 34 to 1892 bp [17]. Mutations in *ADD1* have been shown to be associated with both human and rat hypertension [11, 20, 21]. Human *ADD2* is localized on chromosome 2p13-p14 and has 13 exons [22, 23], whereas human *ADD3* is on chromosome 10q23.2-24.3 and composed of at least 13 introns and 14 exons spanning over 20 kb [24]. *ADD1*, *ADD2*, and *ADD3* are highly homologous with rats, with 94.3%, 91.7% and 91.9% amino acid sequences similar to those of rats, respectively [17, 22, 24].

## 3. Function of Adducin

### 3.1. Adducin Is Essential for the Formation and Stabilization of Membrane Cytoskeleton

Previous studies have shown that adducin promotes the binding of spectrin to actin filaments and is concentrated at the cell-cell contact sites in epithelial cells [25]. It selectively binds to the spectrin-actin complex at the end of its *α* and *β* subunits with a significantly higher affinity than that of either spectrin or actin monomer [26]. Electron microscope confirmed that adducin also contributes to the formation of a complicated meshwork of spectrin and spectrin-actin complexes and the polymerization of actin filaments [25]. This function is regulated by Ca^2+^/calmodulin, protein kinase A and C, and Rho kinase and needs MARCKS-related domain [25, 27–32]. Previous reports indicated that there were two linkages between membrane skeleton and lipid bilayer: band 3-ankyrin-*β*-spectrin and glycophorin C-protein 4.1-*β*-spectrin [33–35]. Anong et al. in 2009 demonstrated that adducin formed a bridge of band-3-adducin-spectrin to consolidate the stabilization of the membrane [35]. Additionally, adducin can also inhibit capping of the fast-growing ends of actin filaments as an actin-capping protein. In this way, adducin could prevent addition or loss of actin subunits and make it easier to bundle actin filaments, as found by Kuhlman and Fowler in 1996 [36, 37].

### 3.2. Adducin Is Involved in the Process of Cell Signal Transduction and Ionic Transportation

Adducin, an in vivo substrate for PKC, PKA, and Rho-associated kinase [27–30], gets involved in cell signal transduction. Moreover, adducin also interacts with other components of membrane skeleton and various membrane proteins to exert effects on ionic transport, particularly with Na^+^ transportation, for instance, the epithelial Na^+^ channels, Na^+^-H^+^ exchange, Na^+^-Li^+^ countertransport, Na^+^-K^+^-Cl^−^ cotransport, and be associated with human EH [6, 38–40]. The adducin gene regulates blood pressure mainly by affecting the activity of the Na^+^-K^+^-ATPase and changing the reabsorption of sodium by the renal tubules [6, 41, 42]. Point mutations in *α*- or *β*-adducin can lead to hypertension as the phosphorylation pattern changes from tyrosine kinase to PKA site [43]. It has been confirmed that mutated *α*-adducin variants have been shown to interact with the Src-SH_2_ domain (Src homology 2), increasing Src activity and Src-dependent Na^+^-K^+^-ATPase phosphorylation and activity. Rostafuroxin, a new antihypertensive drug, blunted this interaction and disrupted Src activation and Na^+^-K^+^-ATPase phosphorylation, resulting in blood pressure normalization in the hypertensive rats [44–46].

## 4. Current Status of the Association between Adducin Family Gene Polymorphisms and EH

Recently, many studies focused on the polymorphisms of adducin family genes and their correlation with EH. Currently, three major loci were highlighted, i.e., Gly460Trp of ADD1, C1797T of ADD2, and A386G of ADD3. However, no clear consensus has been reached on the three major loci and EH, and the relationships remain inconsistent [47–51]. An overview of recent advances on the association between EH and the three main loci is given in the following.

### 4.1. *α*-Adducin Gly460Trp Polymorphism with Hypertension

*α*-Adducin has long been controversial as a risk factor for hypertension. In 1997, Cusi et al. reported for the first time that *α*-adducin Gly460Trp polymorphism is related to EH, especially salt-sensitive hypertension by affecting sodium balance, and suggested that adducin gene could be thought to be one of the candidate genes of EH [21]. Later, Tamaki et al. reached a similar result in the Japanese population; they found that the genotype frequency of Gly460Trp polymorphism and plasma renin activity were significantly different in the normotensive, borderline, and hypertensive groups and that the 460Trp allele might be associated with hypertension, especially the low renin-type hypertension [52], which to some extent supported the result of Cusi's study. In 2007, Nakamura et al. conducted a large-scale community-based research, involving 4,640 participants, including 2,414 subjects with hypertension and 2,226 normal controls, and the results showed that *α*-adducin Gly460Trp polymorphism is associated with high blood pressure and homozygous mutant risk was 1.6 times that of homozygous wild type (OR: 1.6, 95% CI: 1.3–1.9), and they found *α*-adducin Gly460Trp polymorphism can act as an independent risk factor of hypertension in Japanese population [53]. Watanabe et al. carried out a cohort study on Japanese people with normal blood pressure for 12 years recently and found that four SNP sites of different genes, including ADD1 Gly460Trp, were related to EH, and could independently predict the risk of hypertension progression after multiple logistic regression correction [54].

However, cumulative case-control studies came to paradoxical results that the association between *α*-adducin Gly460Trp polymorphism and hypertension varies among ethnic groups [47, 55–57]. All these studies have demonstrated racial differences in genetic polymorphisms associated with EH. Additionally, a Swedish cohort study, including 3,827 subjects with hypertension and 2,178 with normal blood pressure, concluded that *α*-adducin Gly460Trp polymorphism might have few functions in the maintenance of blood pressure and contribution to high blood pressure unless in combination with gender and body mass index (BMI) [58]. Furthermore, the pathological effect of *α*-adducin Gly460Trp polymorphism as a hypertensive disease susceptibility gene in the Russian population was only influenced by environmental factors [59]. These studies suggested that the association of genetic polymorphisms with hypertension is not only related to differences in race and environment but also related to the clinical or biological characteristics of individual subjects. In 2010, two studies about the association between *α*-adducin Gly460Trp polymorphism and EH had reported that there is no association between *α*-adducin Gly460Trp polymorphism and EH in general or in any of the subgroup [51, 60]. It is speculated that the reason may be that the meta-analysis failed to include all the current studies about the association between the polymorphism of *α*-adducin Gly460Trp polymorphism and hypertension. The results of two recent meta-analyses support the hypothesis that T-allele carriers had a higher risk of developing EH in Asian populations, but there was no exact correlation between blacks and whites, highlighting significant ethnic differences in *ADD1* genes [61, 62]. Taken together, the evidence linking *α*-adducin Gly460Trp polymorphism with hypertension is still scanty.

### 4.2. C1797T *β*-Adducin Polymorphism with Hypertension

By now, the association between *α*-adducin Gly460Trp polymorphism and EH has many reports, but the study on the C1797T *β*-adducin polymorphism with hypertension is still in the incipient stage and has many controversies. Wang et al. randomly selected 2,272 Caucasian subjects in northern Belgium to study whether the C1797T polymorphism of the *β*-adducin gene was associated with the risk of hypertension. They found the 1797T allele of the *β*-adducin gene is associated with increased risk of hypertension in postmenopausal women and users of oral contraceptives, especially in woman carrier of the mutated *α*-adducin Trp allele [63], suggesting that the two genes may have potential interactions with each other. A study by Tikhonoff et al. [64] has confirmed that the *β*-adducin C1797T allele may be associated with increased blood pressure in populations with high salt intake. However, Zhou et al. reported that the study of the Yi and Hani ethnic groups with the lowest incidence of EH in China and found that there was no distribution of mutant homozygotes (TT) in both the case group and the control group of the two ethnic groups, and the T allele mutant of *β*-adducin was very low [65], suggesting that the lack of C1797T polymorphism in the Yi and Hani ethnics in China may not be related to the onset of EH. Additionally, Kato et al. discovered a new SNP locus C/A variant of the *β*-adducin gene (rs3755351). The experimental results show that it has a significant correlation with EH, but after a strict Bonferroni correction, the conclusion became negative [66]. Therefore, at the present stage, larger sample sizes and high-quality researches are needed.

### 4.3. *γ*-Adducin A386G Polymorphism with Hypertension

Apart from C1797T *β*-adducin polymorphism, some studies have reported the connection of *γ*-adducin A386G polymorphism with EH, usually combined with the polymorphic loci of ADD1 and ADD2. In 2005, Cwynar et al. studied European Caucasians and found that, in *α*-adducin Trp allele carriers, the increase in peripheral and central pulse pressure was associated with the *γ*-adducin 386G allele, suggesting that two genes may have an epistatic effect that is consistent with the heterodimeric structure of the cytoskeletal protein and its influence on transmembranous sodium transport [67]. In the same year, Lanzani et al. studied the association of the adducin family gene polymorphism with EH and verified the presence of epistatic effects among mutated Trp ADD1 allele and ADD3 G allele. After a combined analysis of the mutant population of ADD1 and ADD3, it was concluded that there was a high correlation with EH [68]. However, in 2006, Chinese scholars reported that there is no 386G allele distribution in the Yi and Hani populations and that the A386G mutation of *γ*-adducin may not be an important determinant associated with EH [65]. Due to the lack of separate studies on the association between *γ*-adducin gene polymorphism and EH, the combined analyses of ADD2 and ADD3 gene variants are also scarce, so the association with blood pressure variability or hypertension is not fully affirmed. Large-scale and in-depth studies are still needed in different populations.

Studies have found that diuretics (especially hydrochlorothiazide, HCTZ) have a better antihypertensive effect in hypertensive patients with adducin gene mutation than nonmutated patients, which indirectly confirmed that adducin gene variation is closely related to volume-dependent hypertension and may be a useful predictor of the antihypertensive effect of diuretics [21, 69, 70]. However, the study by Suonsyrjä et al. found no correlation between adducin gene mutation and EH, presumably because the subjects enrolled in the study were all patients who received antihypertensive drugs and only had a 30-day washout period, so the interference of previous treatment effects on the experiment cannot be excluded [71]. Davis et al. also did not find a predictive effect of the Gly460Trp allele mutation on diuretics, which may be masked by other antihypertensive methods, such as lifestyle adjustments [72]. Schelleman et al. reported a large observational study involving 3,025 hypertensive patients. The results showed that the *α*-adducin Gly460Trp polymorphism had no significant association with the antihypertensive effect of diuretics, but the study did not rule out possible bias and confounding factors and the credibility of the results remains to be evaluated [73]. Due to the lack of relevant research in this field, more high-quality, large-sample studies are needed to further clarify the relationship between adducin family gene polymorphism and the antihypertensive effect of antihypertensive drugs.

## 5. Prospects

Adducin is an important candidate gene for EH though it still faces many controversies. Previous studies had reached inconsistent results; we speculate that there are several reasons as follows: (1) Racial difference: this is because essential hypertension is a highly genetic heterogeneous disease associated with multiple factors, and the majority of studies have found that the onset of essential hypertension is related to the alpha-adducin gene polymorphism mainly in Asian population. (2) Sample size: the small sample size of most case-control studies may be one of the potential limitations. (3) Gene-environmental interactions: most studies do not take into account environmental factors such as geographical location, climate, diet, and lifestyle, all of which are associated with the risk of hypertension. Hence, it is an urgent need to identify gene-environmental interactions in the future. For these reasons, we believe that even if the results are controversial, studies about ADD gene polymorphism will help guide the discovery of the pathogenesis and therapeutic targets of hypertension in the future. As mentioned above, multiple SNPs in adducin family gene are related to the onset of EH, and some new gene mutation loci have been discovered and verified [74, 75]. These studies are helpful to elucidate the genetic mechanism of EH and provide a newer and stronger theoretical basis and practical evidence for the prevention, diagnosis, and treatment of EH. As is shown in the case of rostafuroxin, a newly developed antihypertensive agent targeted endogenous ouabain and mutant adducin and downregulates the Src-epidermal growth factor receptor- (EGFR-) dependent signaling pathway, which leads to the phosphorylation and activation of Na^+^-K^+^-ATPase and ERK tyrosine, thereby selectively inhibiting the activity and expression of renal tubular Na^+^-K^+^-ATPase, suppressing sodium reabsorption in renal tubules, and lowering blood pressure. As described above, a 30-day washout period is not enough to remove the influence of previous treatment. Hence, Lanzani et al. conducted clinical trials in newly discovered and untreated patients to determine whether genetic profile could predict the response to the pharmacological treatment with rostafuroxin [76]. They found in these clinical studies that the relevance of adducin gene variants is able to predict the response to the new antihypertensive medication. Additionally, rostafuroxin reduced blood pressure in these patients who responded to rostafuroxin while inhibiting hypertension-related organ damage, thereby reducing cardiovascular risk factors. Given their involvement in essential hypertension, adducin gene variants represent attractive therapeutic target and it is reasonable to believe that they can be used to predict the response to rostafuroxin. Our collective hope is that by identifying positive genes for EH, we will be able to better predict those at risk and, perhaps most importantly, develop new treatments and use them in more precise ways.
